# Factors influencing the self-awareness of falls in hospitalized older adults: a Q method study

**DOI:** 10.3389/fpubh.2025.1728695

**Published:** 2026-01-14

**Authors:** Tianxin Miao, Ke Chen, Yingna Zhao, Dianli Han, Liran Duan, Haiyue Gu, Lan Zhang, Dan Li, Ying Yao

**Affiliations:** 1 Tianjin Medical University General Hospital, Tianjin, China; 2 Department of Emergency Medicine, Tianjin Medical University General Hospital, Tianjin, China

**Keywords:** falls, hospitalized, influencing factors, Q methodology, self-awareness

## Abstract

**Background:**

Recognizing the risk of falls is crucial for fall prevention among hospitalized older adults. However, awareness of fall risk in this population is generally poor. There is also a lack of research exploring the factors influencing their fall risk awareness from the patients’ subjective perspective.

**Methods:**

Hospitalized patients aged 60 years or older were recruited from a tertiary hospital in Tianjin, China. Participants completed a Q-sorting task in which they ranked 34 statements regarding factors potentially influencing fall awareness. Following the sorting activity, post-sorting interviews were conducted to explore the reasoning behind their rankings and to further assess the factors shaping fall self-awareness. Principal component factor extraction was employed to analyze differences in statement rankings across participants. Additionally, descriptive analysis of the qualitative interview data was performed to elucidate the underlying reasons for these observed differences.

**Results:**

The analysis of the ranked results of 15 patients (mean age 68.5 ± 7.8 years; 73.3% male) revealed three statistically independent factors, representing distinct types of influences on fall self-awareness among hospitalized older adults: (1) Fall-Derivative Type (*n* = 7), (2) Family-Oriented Type (*n* = 2) and (3) Healthy State Type (*n* = 6). Positive influencing factors included the level of attention to fall prevention, self-care ability, and the type and number of diseases. In contrast, negative factors were associated with literacy levels and social.

**Conclusion:**

Falls self-awareness in older inpatients is influenced by multiple factors and varies significantly based on individual cognition. Patients can be categorized into three types: Fall-Derivative, Family-Oriented, and Healthy-State. To enhance falls self-awareness and reduce fall rates, healthcare professionals should tailor their health education by first identifying the patient’s specific type and then providing personalized guidance accordingly.

## 1 Background

Falls represent the second leading cause of accidental injury mortality and the third leading cause of chronic disability worldwide (1). According to the survey, the prevalence of falls in the world’s older people was 26.5%. (2). The comprehensive incidence of falls among the older adults after hospitalization is 14% (3). These incidents are often associated with serious physical injuries—such as fractures, disability, and death—as well as increased healthcare costs (4–7). One study indicated that regardless of whether physical injury occurs, a fall during hospitalization leads to substantial costs, likely due to extended hospital stays (8). This issue is particularly pronounced in geriatric care units, where inpatient falls are frequent due to a combination of recurrent intrinsic and extrinsic risk factors among patients. Importantly, it is said that those who fall and are not harmed often suffer the negative consequences of that fall (2).

A study by Kantow et al. (9) revealed that many hospitalized older adults do not perceive themselves as being at risk of falling, or are simply unaware of their risk, which poses a significant barrier to fall prevention. Understanding the factors that influence older inpatients’ self-awareness of fall risk can inform targeted prevention strategies, outreach programs, and communication with this population (10). Self-awareness of falls refers to the subjective degree of the patient’s perception of the risk of falling and is the basis for fall prevention (11, 12). Higher levels of self-awareness are associated with greater willingness to participate in fall prevention programs and better adherence, thereby reducing the likelihood of falls (10). Existing studies on this topic are cross-sectional and indicate that fall self-awareness is related to factors such as age, fall-related injuries, and fear of falling (13, 14). However, few studies have explored these influencing factors from the patients’ own perspective. Identifying factors that affect self-awareness from the patient’s viewpoint can help determine the priority of these factors as perceived by patients. This, in turn, would enable healthcare providers to design more targeted educational interventions, enhance patients’ self-awareness levels, and improve the efficiency of fall prevention efforts (15).

To explore which factors are subjectively perceived by older inpatients as influencing their fall self-awareness, this study employed the Q-methodology—a mixed-methods approach suitable for investigating subjective viewpoints on value-laden topics (16). This method integrates the strengths of qualitative and quantitative analysis while centering the perspectives of patients (17, 18). The aim of this study is to identify the factors influencing fall self-awareness as judged subjectively by hospitalized older adults, thereby providing a reference for developing strategies to enhance their self-awareness and support fall prevention.

## 2 Methods

### 2.1 Design

**Figure 1:**
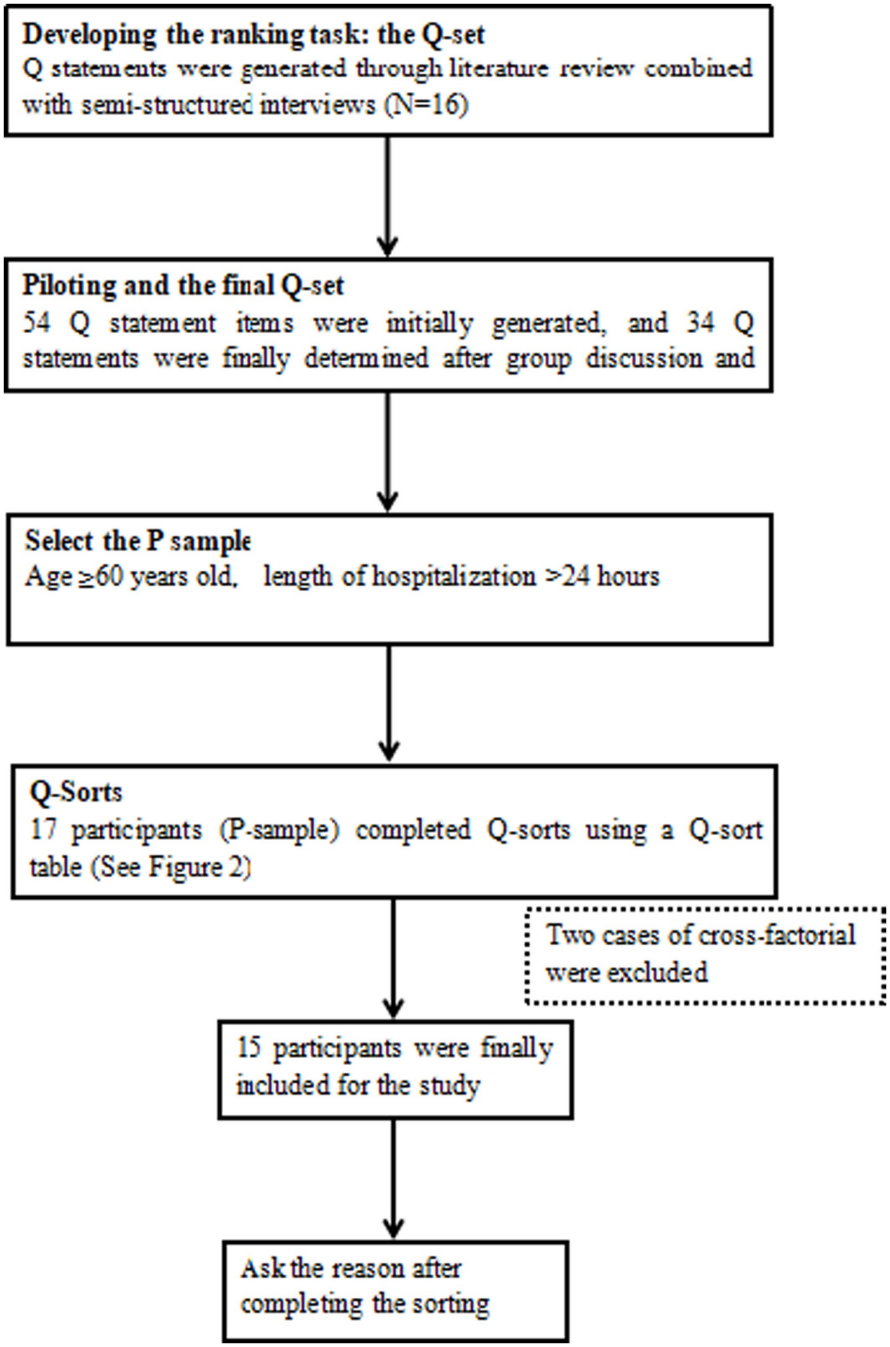
The process of the Q method.

Q-methodology was used in this study. It was developed to explore a person’s viewpoints (e.g., feelings, perspectives, and attitudes) on a given topic, systematically identify the similarities and differences between these viewpoints, and probe the reasons behind them (19, 20). Therefore, this methodology is considered suitable for patients’ subjective judgment of the influencing factors of fall self-awareness. This study is not registered as it is not part of a clinical trial. For the specific operation process, see Figure 1.

### 2.2 Participants

In this Q-methodology study, participants were purposively recruited from inpatient wards during their hospital stay to capture diverse perspectives. Hospitalized patients aged ≥60 years with a length of stay >24 h were identified by ward staff. Those with severe dementia, communication barriers, or major mental illness were excluded. All enrolled participants provided written informed consent. Data on age, sex, education level, and hospitalization length were collected.

### 2.3 Procedures

The processes of statement development, data collection and analysis and reporting for this study were conducted in line with published guidelines for Q-methodology studies (19, 21, 22).

#### 2.3.1 Developing the ranking task: the Q-set

The first step in Q-methodology is to develop a concourse—a comprehensive set of statements representing the “universe of opinions” on a given topic (23). To construct the initial Q-set, the research team employed a combination of literature review and one-on-one interviews with hospitalized older patients. From September to October 2024, a total of 16 participants were recruited, with the sample size determined by reaching data saturation. Before the interview, the participants were informed of the study’s purpose and procedures and the use of recording; written informed consent was obtained. The interviews were conducted in a quiet private conference room by two nursing graduate nurses trained in qualitative research: one led the discussion using the outline, and the other recorded and supplemented, with both interviewers avoiding leading language. Verbatim transcription was completed within 24 h, and the transcripts were returned to the participants for checking.

The interview guide included the following questions:

1. What do you think are the harms associated with falls in older patients?
2. What types of people do you consider to be at risk of falling?
3. During your hospital stay, do you consider yourself at risk of falling? Why or why not?
4. What kinds of people do you think need fall prevention?
5. Do you think you need fall prevention? Why or why not?
6. Have you ever experienced a fall? If so, how did it occur?

Participants were primarily recruited from internal medicine and emergency wards for interviews. Their medical conditions covered a range of diseases, including respiratory, circulatory, urinary, digestive, endocrine, and peripheral tissue disorders. Since the attention span of older patients is relatively short, the interview time was kept brief. Each interview lasted approximately 15–20 min, and data saturation was achieved. Among the 16 participants, eight had experienced a fall within the past year, while the other had not. The sample consisted of nine males and seven females, with the following age distribution: five aged 60–70 years, 10 aged 71–80 years, and one aged 81–90 years. Educational backgrounds varied: two had primary school education, six had junior high school education, three had high school education, and five had college education. In terms of residential background, 10 participants were from rural areas and six from urban areas.

#### 2.3.2 Piloting and the final Q-set

After initial development, a preliminary Q-set of 54 statements across four themes was extracted. The themes include: biophysiological factors (directly related to an individual’s physical function and health status), psychological and cognitive factors (pertaining to emotions, cognition, beliefs, and behavioral awareness), social and environmental factors (covering the individual’s physical environment, social support, cultural and economic background), and fall-specific factors (referring specifically to the attributes and direct consequences of the fall incident). Through group discussions and consultations with three experts in clinical nursing and nursing management (two with master’s degrees and one with a doctoral degree, all holding the title of Chief Nurse), statements with overlapping meanings were consolidated. The Q-set was ultimately refined to 34 statements, which falls within the acceptable range of 25–80 statements (16), see Supplementary Table S1.

#### 2.3.3 Sample size, participants and recruitment

Q-methodology studies generally have smaller sample sizes, similar to those found in qualitative studies. The aim of sampling in Q-methodology is to include sufficient individuals to establish the existence of different viewpoints, not to determine the proportion of the population holding each view (16). The recommended sample size is 4–6 participants per anticipated viewpoint type. Given that this study anticipates 3–4 distinct viewpoints, the projected P-set sample size is 12–24 participants (22). For the final Q set of 34 statements, a P set of 17 participants was recruited, resulting in an effective sample size of 15 after data validation.

#### 2.3.4 Data collection procedures—“Q-sorting”

**Figure 2:**
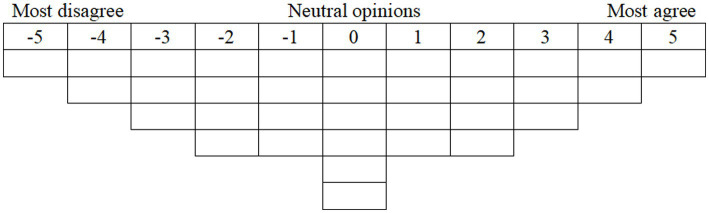
Forced-choice frequency distribution in the Q-sorting process.

During November and December 2024, study participants sorted the 34 statements derived from an iterative thematic analysis, ranking them from least to most important based on the instruction: “What factors do you think have the greatest impact on self-awareness of falling?” After the order of statements was randomized, participants were asked to place the one statement they agreed with the most (+5) in the far-right column and the statement they disagreed with the most (−5) in the far-left column. Participants then repeated the procedure for the next column (+4 and −4) by selecting two agreed and two disagreed statements from the remaining statements. This procedure was repeated until the Q-sort grids were all completely filled (Figure 2). Participants were allowed to adjust their sorting decisions at any time until they indicated no further changes were desired. After sorting was complete, the researcher asked participants to explain their ranking rationale, with particular attention to the reasons for their most and least agreed statements, for subsequent analysis. It took a total of 30–40 min to complete.

### 2.4 Statistical analysis

Statistical analysis of the data was performed using PQ Method 2.35, PASW 18.0, and KADE 1.2.1 software (24). The data were first entered into PQ Method 2.35 to prevent duplication or omission. Each participant’s ranking of the statements was transformed into an array of numerical data ranging from −5 to +5 according to their placement in the columns of the score sheet. Subsequently, PASW 18.0 was employed for statistical description: measurement data were expressed as mean ± standard deviation, while categorical data were summarized as frequencies, percentages, or rates. The Kaiser–Meyer–Olkin (KMO) measure and Bartlett’s test of sphericity were conducted to verify that the data met the prerequisites for factor analysis. Finally, KADE 1.2.1 was used to perform principal component analysis for factor extraction. The analytical process included the following steps (25): (1) Factor extraction. Principal component analysis was applied to extract factors, retaining those with eigenvalues >1 and a cumulative variance contribution ≥40%. To facilitate interpretation and labeling, the extracted factors were rotated using the varimax method, which enhances the clarity of factor classification. (2) Calculation and classification of factor loadings. Factor loadings reflect the strength of the correlation between participants (P-sample) and the factors. Higher absolute values of factor loadings indicate greater statistical significance and a stronger association between the participants and the corresponding factors. The threshold for a statistically significant factor loading was determined by dividing the multiplier for the desired significance level by the square root of the number of statements in the Q set (multipliers: 3.29 for *p* < 0.001; 2.58 for *p* < 0.01; 1.96 for *p* < 0.05) (22). For a factor to be considered defined, it was required to be loaded by at least two participants. Any participant whose loadings reached significance on two or more factors was considered confounded and therefore excluded from subsequent analyses. (3) Calculation of factor scores. Following factor extraction and rotation, the KADE 1.2.1 software automatically calculated the factor scores for each statement (Q-sort). These scores were then ranked to form a factor array, resulting in an idealized ranking for each distinct viewpoint. Each factor was interpreted using both its factor array and the qualitative data from participants who loaded significantly on that factor. In this context, a factor represents a shared perspective on the elements influencing fall-related self-awareness. This perspective is an abstract construct that encapsulates the common viewpoint shared by participants whose statement rankings were sufficiently similar to load significantly on the same factor.

### 2.5 Ethical considerations

This study was approved by the Ethics Committee of Tianjin Medical University General Hospital, on 23 January 2025 (approval No.: IRB2025-YX-039-01).

## 3 Results

### 3.1 Characteristics of participants

A total of 15 participants aged 68.53 ± 7.836 years were recruited; 73.3% (*n* = 11) of them were men, as detailed in Table 1. In this study, the KMO measure was 0.797, and Bartlett’s test of sphericity yielded an approximate *χ*^2^ value of 397.734 (*p* < 0.001), indicating that the data were suitable for factor analysis. Using the formula multiplier/√*n*, the significance threshold was calculated as 3.29/√34 ≈ 0.564. Based on the criterion, participants P10 and P12 were removed due to cross-loadings. Consequently, 15 participants were retained and categorized into three distinct factors, as presented in Table 2. Three perspective factors were derived from the Q-sorting results (Table 3). The explained variance by the three perspectives were 50% (eigenvalue = 7.441), 10% (eigenvalue = 1.516), 8% (eigenvalue = 1.205). The total explained variance was 68%.

**Table 1:** Socio-demographics characteristics with the three factors.

Variables	Factor 1 (*N* = 7)	Factor 2 (*N* = 2)	Factor 3 (*N* = 6)	Total (*N* = 15)
Age (year, Mean ± SD)	72.85 ± 7.471	62.50 ± 3.536	65.50 ± 7.120	68.53 ± 7.836
Gender				
Male	5 (71.4%)	1 (50%)	5 (83.3%)	11 (73.3%)
Female	2 (28.6%)	1 (50%)	1 (16.7%)	4 (26.7%)
Education				
Primary school	1 (14.3%)	1 (50%)	0 (0%)	2 (13.3%)
Middle school	3 (42.9%)	0 (0%)	1 (16.7%)	4 (26.7%)
Professional/high school	2 (28.6%)	1 (50%)	4 (66.7%)	7 (46.7%)
College/university	1 (14.3%)	0 (0%)	1 (16.6%)	2 (13.3%)
Length of hospitalization (days)	5.14 ± 2.734	21.00 ± 9.899	5.33 ± 1.966	7.33 ± 6.510
1–3	2 (28.6%)	0 (0%)	1 (16.7%)	3 (20%)
4–7	4 (57.1%)	0 (0%)	4 (66.7%)	8 (53.3%)
8–10	1 (14.3%)	0 (0%)	1 (16.7%)	2 (13.3%)
>10	0 (0%)	2 (100%)	0 (0%)	2 (13.3%)
Marital status				
Single	2 (28.6%)	0 (0%)	0 (0%)	2 (13.3%)
Married	5 (71.4%)	2 (100%)	6 (100%)	13 (86.7%)
Place of residence				
Town	6 (85.7%)	0 (0%)	3 (50%)	9 (60%)
Countryside	1 (14.3%)	2 (100%)	3 (50%)	6 (40%)
Fall experience				
Yes	2 (28.6%)	0 (0%)	0 (0%)	2 (13.3%)
No	5 (71.4%)	2 (100%)	6 (100%)	13 (86.7%)
Primary disease category				
Respiratory system diseases	3 (42.9%)	0	3 (50%)	6 (40%)
Cardiovascular diseases	1 (14.3%)	0	1 (16.7%)	2 (13.3%)
Digestive system diseases	3 (42.9%)	0	2 (33.3%)	5 (33.3%)
Oncological diseases	0	2 (100%)	0	2 (13.3%)

**Table 2:** Distribution of participants on three factor classifications.

Q-sort	Factor group	Factor 1	Factor 2	Factor 3
P8	F1-1	0.858^*^	0.1231	0.0125
P9	F1-2	0.6953^*^	0.0865	0.4566
P7	F1-3	0.6438^*^	0.062	0.4758
P2	F1-4	0.6372^*^	−0.2657	0.235
P3	F1-5	0.6323^*^	0.4745	0.3332
P6	F1-6	0.6125^*^	0.1065	0.3856
P4	F1-7	0.6055^*^	0.1245	0.5415
P13	F2-1	−0.0017	0.841^*^	−0.0528
P16	F2-2	0.105	0.7052^*^	0.4941
P15	F3-1	0.1671	0.1853	0.8197^*^
P17	F3-2	0.3671	0.0308	0.8128^*^
P11	F3-3	0.3712	0.0487	0.809^*^
P1	F3-4	0.3079	0.3842	0.694^*^
P5	F3-5	0.1446	−0.0289	0.6921^*^
P14	F3-6	0.3135	0.1222	0.68^*^

* *p* < 0.001.

**Table 3:** Types of different influencing factors, their eigenvalues and explanatory variation.

Factor	Eigenvalues	Explained variance (%)	Cumulative explained variance (%)
1	7.441	50	50
2	1.516	10	60
3	1.205	8	68
4	0.967	6	74
5	0.751	5	79
6	0.690	5	84
7	0.556	4	88
8	0.486	3	91

### 3.2 Consensus about influencing factors of self-awareness of falls

**Figure 3:**
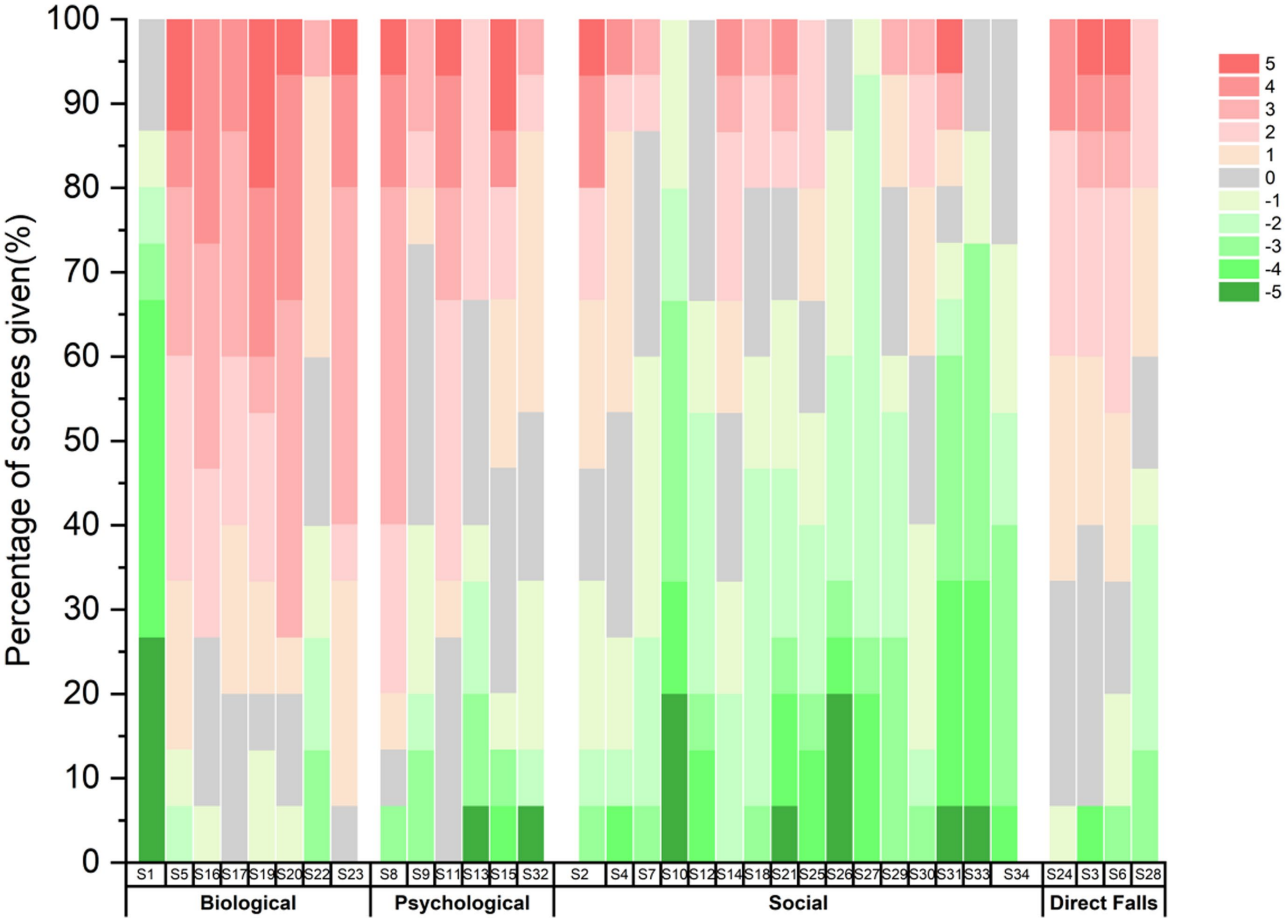
Distribution of the overall scoring of statements.

Figure 3 shows the frequency distribution of the rankings of the 34 statements by the 15 participants. The colors indicate the level of agreement (*y*-axis) of the participants with the statements (*x*-axis). A +5 (dark red, agree) indicates that the participant placed the statement in the +5 box on the score sheet, while a −5 (dark green, disagree) represents the opposite.

According to Figure 3, the Q-statements S23 (“The type and number of diseases affects one’s self-awareness of falls”), S10 (“Literacy affects one’s self-awareness of falls”; ranked −4, −5, −4 across factors), and S27 (“Fall Prevention Policy can affect one’s self-awareness of falls”; ranked −2, −3, −2 across factors) showed the highest level of consensus. This consistency suggests that disease type and number are factors they consider to be associated with self-awareness of falls, while education and fall prevention policies are two factors that are overlooked by patients. The values in parentheses represent the Q-sort scores of each statement across the three factors.

Based on the results of principal component analysis (Table 3), a total of three factors were extracted in this study. The corresponding Q sample sizes are: 7, 2, 6.

### 3.3 Fall derivative type

Seven participants were mapped to Factor 1, comprising five males and two females. The corresponding subjects were P2, P3, P4, P6, P7, P8, P9. As shown in Table 1, these patients had a mean age of 72.85 ± 7.471 years and had a shorter duration of hospitalization compared to those in Factor 2 and Factor 3. Two patients with previous falls were categorized in this group. Since these patients primarily based their judgment of fall awareness on concerns related to the fall itself, we named this perspective the Fall-Derivative Type. For example, the q-statement S15 “The level of fall knowledge will affect one’s self-awareness of falls” (Z-score, Q-sort: 1.66^**^, 5) and S24 “The unpredictability of falls can affect one’s self-awareness of falls” (1.37^**^, 4), suggesting that hospitalized older patients perceive fall-related content as a major influencing factor, see Supplementary Table S2.

### 3.4 Family-oriented type

Two participants were mapped to Factor 2, including one male and one female. The mean age of these patients was 62.50 ± 3.536 years. A notable feature is that they were both cancer patients, had the longest hospitalization time, and lived in rural areas. They all considered their own risk of falling out of concern for their families and children, so they were named Family-Oriented Type. Among them, S31 “Economic conditions can affect one’s self-awareness of falls” (2.03^**^, 5) and S21. “The number of children can affect one’s self-awareness of falls” (1.56^**^, 4) are the most important influencing factors.

In addition to the uniform negative factors, the main statements from which participants in this group dissented, differing from their peers, were: “The degree of attention paid to fall prevention will affect one’s self-awareness of falls” (−0.94^**^, −3); “The level of fall knowledge will affect one’s self-awareness of falls” (−1.25^**^, −4). The low ranking of these statements indicates that they do not value the impact of the fall itself.

### 3.5 Healthy state type

Six participants were mapped to Factor 3, of which five were male and one was female. The corresponding subjects are P1, P5, P11, P14, P15, P17. The patients in this group had a mean age of 65.50 ± 7.120 years, a relatively long hospital stay, a high level of education, and no fall experience. They are more concerned about their condition and assess their risk of falls based on their physical health. Hence the name Healthy-State Type. Such patients believe that the judgment of whether they are at risk of falling is based on their current physical condition, stating that “changes in their condition will affect self-awareness of falls” (1.84^**^, 5) and “Physical fitness can affect one’s self-awareness of falls” (1.74^*^, 4) are the main influencing factor expressed by this type of patient.

## 4 Discussion

Discussing fall self-awareness with hospitalized older adults may enhance their recognition of fall risks and encourage proactive preventive behaviors. This study found that older patients’ understanding of self-awareness of falls is primarily limited to the basic notion of “staying alert to falls.” However, self-awareness of falls is actually a multidimensional concept that includes not only the subjective perception of fall risk but also the objective self-assessment of individual risk and the initiative to take preventive actions (12).

The results indicate that older patients generally consider the number and types of diseases to be the main factors influencing their self-awareness of falls, a finding which aligns with previous research (26). Patients with less comorbidity often struggle to associate fall risk with themselves. As the number of conditions increases, multiple diseases can have cumulative or synergistic effects on physical impairment. This not only leads to declines in central nervous system control, coordination, and reaction capacity but may also exacerbate functional impairment and reduced balance due to disease-specific effects. Examples include movement disorders caused by neurological conditions, decreased environmental perception due to sensory system diseases, or sudden alertness collapse from acute episodic illnesses (27, 28). Moreover, a higher disease burden is often accompanied by polypharmacy. Side effects of medications—such as drowsiness from sedatives or orthostatic hypotension from antihypertensive drugs—interact with the diseases themselves, collectively increasing fall risk (29). Additionally, certain conditions like visual or hearing impairment directly affect older adults’ ability to perceive their surroundings, making it difficult to identify potential hazards. These factors, within the context of multimorbidity and polypharmacy, further elevate the likelihood of falls (27).

However, it is noteworthy that participants generally denied the influence of education level and fall prevention policies on self-awareness of falls—a view that contrasts with existing quantitative evidence (30–33). Most interviewees considered self-awareness of falls to be primarily a matter of personal awareness, rather than being influenced by formal education or policy. Research evidence (9, 31) suggests that patients with lower education levels may lack health-related knowledge, have difficulty objectively assessing their own fall risk, and show less willingness to proactively learn about fall prevention, thereby resulting in insufficient fall self-awareness. This observed disregard for the role of education among participants may itself constitute a barrier to improving self-awareness.

In recent years, relevant authorities in China have increasingly prioritized fall prevention, having developed and implemented a range of preventive measures while integrating fall prevention into the public service system (34). However, surveys indicate that public awareness and acceptance of these policies remain low, partly due to insufficient policy promotion and a general lack of health consciousness (35). This phenomenon has, to some extent, weakened both the social attention to and the effectiveness of fall prevention initiatives.

Accordingly, we recommend that clinical healthcare providers incorporate an assessment of educational level as part of the individual capacity evaluation, alongside emphasizing disease-related factors. Health education should then be tailored based on the patient’s competency. Furthermore, tangible benefits—such as medical insurance coverage and age-friendly home modifications—should be included in educational content to help patients perceive the value of public services in safeguarding their safety. Through such multidimensional health education, it may be possible to improve fall self-awareness among hospitalized older patients and reduce fall risk. These insights offer important implications for refining clinical fall prevention intervention strategies.

### 4.1 Fall derivative type

Patients classified under the “Fall-Derivative” profile perceive a direct connection between their self-awareness of falls and fall-related factors, primarily including the unpredictability of falls and their level of knowledge about fall prevention. This profile represents a considerable proportion of the study sample and is characterized mainly by advanced age and a history of previous falls.

Research indicates that a history of falls is closely associated with the level of knowledge about falls. Patients who have experienced a fall tend to possess a better understanding of fall-related information than those without such a history. This may be because older adults who have experienced a fall often prioritize fall prevention in their daily decision-making, pay more active attention to potential environmental risk factors, and are consequently more motivated to proactively learn about fall-related knowledge (36, 37). At the same time, a history of falls influences older patients’ attitudes toward falling. Those who have never experienced a fall often perceive it as an accidental event, believing that preventive measures are unnecessary before a fall occurs and that taking early action would be futile. Consequently, they may underestimate the need for advance prevention or vigilance (38). For this group, health education needs to emphasize the preventable nature of falls, enhance patients’ understanding, and thereby promote improved self-awareness of falls.

### 4.2 Family-oriented type

Patients categorized under the “Family-Oriented” profile demonstrated a relative neglect of personal fall risk, focusing instead on the potential family distress and caregiving burden that a fall might cause. In this study, this profile was characterized by a longer duration of illness and significantly longer hospital stays compared to other types. Their perspective on factors influencing self-awareness of falls showed a distinct family-oriented tendency, which aligns with previous research (39).

Studies indicate that such patients often strive to maintain a higher level of self-awareness of falls in order to reduce the caregiving burden on their children (8, 9). This tendency stems from two main concerns: first, falls could impose additional medical expenses on their children, increasing the financial burden on the family; second, in single-child families, the absence of sibling support may exacerbate the psychological and emotional pressure on the child. As a result, these patients are more inclined to consider family welfare in their behavior. Unlike the other two profiles, however, these patients tended to overlook the direct impact of falls themselves on self-awareness. Further interviews revealed a cognitive bias: although they self-rated their knowledge of fall prevention as adequate, their actual preventive measures remained limited to basic physical protection, with clear deficiencies in understanding professional aspects such as medication side effects and disease-specific risks. This cognitive limitation reflects a tendency among long-term hospitalized patients to develop “prevention fatigue” and exhibit cognitive rigidity regarding fall prevention (13).

Consequently, interventions targeting this group should aim to reshape these cognitive patterns. Implementing a family-collaborative education model that emphasizes knowledge of medication and disease-related risks, coupled with establishing a family supervision mechanism, could help ensure the implementation of preventive measures. In addition, healthcare providers should pay particular attention to the influence of patients’ financial circumstances on protective behaviors, ensuring that intervention plans balance professionalism and feasibility. Such a multifaceted approach could more effectively enhance fall self-awareness in this patient profile.

### 4.3 Healthy state type

Patients categorized under the “Healthy State” profile were characterized by relatively short hospital stays. Their self-awareness of falls was predominantly influenced by their own health status: when they perceived their health as poor, their attention to falls increased. This result is consistent with studies by Ong et al. (40), which indicates that older persons with objectively poor health may have increased self-awareness of falls, despite having decreased physical function. This heightened awareness may stem from a more realistic appraisal of their functional limitations, prompting greater vigilance.

Conversely, patients in good health may lower their alertness to fall risks due to overconfidence in their own abilities. However, interviews revealed that some older patients had an inadequate perception of their own health status, manifested as overconfidence, a lack of self-protection awareness, or underestimation of the severity of fall risks (41). This may be related to their shorter hospitalization periods. Moreover, the health status of hospitalized older adults can change with disease progression. For such patients, healthcare providers could introduce simple, user-friendly self-assessment tools during fall prevention education, encourage the development of habitual self-health monitoring, and provide timely personalized health guidance. These strategies represent promising interventions for enhancing fall self-awareness in this patient profile.

A key strength of this study lies in its inclusive design. The use of magnetic boards to facilitate the sorting task helped mitigate common participation barriers related to vision or manual dexterity, thereby optimizing the engagement experience and data quality for older patients. Furthermore, this research applies Q-methodology to systematically identify, from the patients’ own subjective perspective, a typology of factors influencing fall self-awareness, thereby providing a novel evidence base for developing targeted health education interventions in clinical practice. In order to facilitate the clinical application of patient typology, a rapid screening method suitable for busy hospital environments can be developed in the future. Healthcare professionals can briefly assess the patient’s perspective through short, targeted questions that align with these three characteristics.

This study has the following shortcomings: first, the enrolled older inpatients were all recruited from a single large tertiary hospital in Tianjin, and the sample was not multi-centered. Consequently, the relatively homogeneous background of the participants may limit the richness and diversity of the perspectives collected. The employed methodology, Q-methodology, is inherently designed to capture individual subjective viewpoints and to “discover” distinct perspectives rather than to ensure their generalizability to a broader population. The small sample size further restricts the generalizability of the findings. Future research should aim to include a larger and more diverse sample. Second, regarding the development of the Q-statements, although they were grounded in a comprehensive literature review and semi-structured interviews, the perspectives of inpatients from other provinces and cities were not incorporated. Therefore, the coverage of potential viewpoints may not be fully comprehensive. Third, during the Q-sorting process, despite the researcher spending considerable time explaining the procedure to participants, the number of statements to be sorted was somewhat large, placing high demands on them. This may have, to some extent, induced fatigue or frustration in participants, potentially leading to biases in the resulting sorts. Finally, this study had a higher proportion of male participants in the sample due to the large number of male patients who actually met the inclusion criteria during the study collection period. Given that gender may influence self-awareness of falls, this imbalance should be considered a potential limitation, as it could affect the generalizability of the findings to female older inpatients. Future studies should aim to include more gender-balanced samples to further validate and extend these findings.

## 5 Conclusion

This study identified three main types of factors that affect the awareness of fall risk in hospitalized older adults, namely fall derivative, family oriented, and healthy state. Different types of patients are concerned about different influencing factors, but the commonality is that demographic factors are often overlooked. This suggests that medical staff can tailor communication by focusing on the factors of concern according to the specific type of hospitalized older adults, thereby improving patients’ self-awareness.

## Data Availability

The raw data supporting the conclusions of this article will be made available by the authors without undue reservation.
