# Supplementary material for: Factors influencing the self-awareness of falls in hospitalized older adults: a Q method study
Source: Front Public Health. 2026 Jan 14;13:1728695. doi: 10.3389/fpubh.2025.1728695 (PMC12846958; doi:10.3389/fpubh.2025.1728695)
Supplement: Supplementary file 1 [file Data_Sheet_1.PDF]

Among these, statements 1–43 were derived from interviews, and statements 44–54 were obtained from literature review.

Table S1 Full set of statements: the Q-set

Relevant statements extracted from the interview/literature review	Q-statements
1. My family will be with me at all times during my hospitalization and I don't think I will have a fall.	1. Inpatient accompaniment can affect one's self-awareness of falls(S2)
2. I've always had a good physique, and I don't think I'm going to fall	2. Physical fitness can affect one's self-awareness of falls(S20)
3. My immunity has decreased	
4. I probably won't be able to get up after I fall	
5. I have had falls, but they have not resulted in injury, so I don't think I need to pay attention to fall prevention	3. Injuries from falls affect one's self-awareness of falls(S3)
6. I'm afraid of burdening my family, so I'll be careful to prevent falls	
7. I'm afraid of causing harm to my body.	
8. I'm worried that I won't be able to hang out with my friends after I fall	
9. Falls will definitely hurt me	4. The comprehensive strength of the hospital will affect one's self-awareness of falls(S12)
10. The hospital I stayed in was very strong and there were no falls	
11. I've gone through hospitalization and have the strength to not have a fall	5. Changes in one's own condition can affect self-awareness of falls(S19)
12. I didn't think about whether or not I'd have a fall.	6. One's own personality affects one's self-awareness of falls(S13)
13. I think I've taken care of falls, so I don't think I'm at risk of falling	
14. I am a careless person	
15. In the hospital, I make it a point to walk slowly so I don't fall.	7. Awareness of fall prevention affects one's self-awareness of falls(S11)
16. The doctors and nurses at the hospital would always remind me, so I didn't think I was at risk of falling	8. The reminders of medical staff will affect their self-awareness of falls(S18)
17. I'm getting older	9. Age affects one's self-awareness of falls(S5)
18. I think 60 years old is young	
19. I felt that the environment during my hospitalization was not good, which could cause me to fall	10. Hospitalization environment affects one's self-awareness of falls(S4)
20. I became familiar with the inpatient environment	
21. There is no risk of falling unless the road surface is uneven or there are obstacles	
22. The environmental safety of the hospital is well done	
23. There is no risk of falling unless the road surface is uneven or there are obstacles	
24. Falling will have unpredictable consequences for me	11. The unpredictability of falls can affect one's self-awareness of falls(S24)
25. Accidental falls can happen to everyone	
26. Falls are an accidental event that cannot be prevented	
27. I've had a fall before	12. History of falls affects self-awareness of falls(S6)
28. My illness predisposes me to falls	13. The type and number of diseases affects

29. The number of chronic diseases ≤ 1, which to a certain extent indicates that the elderly patients with hypertension have better overall health, relatively strong physical function and immune system, and are less troubled by health problems in daily life, so they may lack vigilance about their own health status	one's self-awareness of falls(S23)
30. During my hospitalization, I didn't get out of bed much except to go to the bathroom<br>31. I'm in the hospital and don't go out	14. Getting out of bed activity time will affect one's self-awareness of falls(S14)
32. My neighbor and friend died after a fall and being bedridden for a while	15. Falls by people around you can affect your self-awareness of falls(S25)
33. I know that when I fall, I need to protect my head first<br>34. Fall prevention is just a matter of being careful, how else can you pay attention?	16. The level of fall knowledge will affect one's self-awareness of falls(S15)
35. I just stayed in the hospital because I was afraid of falling.	17. Fear of falling affects one's self-awareness of falls(S9)
36. It is important to take care of falls at all times<br>37. Preventing falls is common sense, so I wouldn't pay too much attention to it	18. The degree of attention paid to fall prevention will affect one's self-awareness of falls(S8)
38. My legs and feet are not good<br>39. My eyesight is not good	19. A physical disability can affect one's self-awareness of falls(S16)
40. My family always reminded me	20. Family reminders affect self-awareness of falls(S30)
41. Falls are inherently rare	21. The probability of a fall will affect one's self-awareness of falls(S28)
42. I need to go out for a checkup.	22. The need to go out for check-ups can affect one's self-awareness of falls(S29)
43. I'm anxious.	23. Self-psychological state affects one's self-awareness of falls(S32)
44. The more educated the patient is, the more he knows and will be aware of fall prevention	24. Literacy affects one's self-awareness of falls(S10)
45. Patients with good economic conditions pay more attention to their health and are more aware of prevention than those with poor financial conditions	25. Economic conditions can affect one's self-awareness of falls(S31)
46. Talking to others has given me a better understanding of falls and increased my alertness to the risk of my own falls	26. Social interaction can affect one's self-awareness of falls(S26)
47. Patients who are self-care well generally do not think that they have fallen	27. Self-care ability can affect one's self-awareness of falls(S17)
48. Patients who sleep less than 7 hours a day may underestimate their risk of falling	28. Sleep status can affect one's self-awareness of falls(S22)
49. Urban areas may have higher accessibility and utilization of healthcare services relative to rural areas, and therefore have differences in fall alertness	29. The place of residence affects one's self-awareness of falls(S33)
50. The low degree of family care means that patients lack sufficient support and supervision in their lives, which leads to a decrease in patients' attention to their own health and fall risk.	30. Family care affects one's self-awareness of falls(S7)
51. Elderly people with a small number of children may be concerned about causing trouble for their children and thus value their	31. The number of children can affect one's self-awareness of falls(S21)

own fall risk	
52. Older adults with frequent health checkups have better fall risk perceptions	32. Fall Prevention Policy can affect one's self-awareness of falls(S27)
53. Men and women have different perceptions of fall risk	33. Gender affects one's self-awareness of falls(S1)
54. My relationship with the nurse has allowed me to increase my perception of the risk of falling	34. The relationship between caregivers and patients can affect their self-awareness of falls(S34)

Table S2 List of Q-statements and factor Q-Sort values associated with the three perspectives

Statement Number	Factor 1			Factor 2			Factor 3		
	Z-score	Score	Rank	Z-score	Score	Rank	Z-score	Score	Rank
S1	-1.67	-5	34	-0.77*	-2	25	-2.04	-5	34
S2	0.82	2	9	0.47	1	13	-0.26	-1	21
S3	0.28	0	17	0.62	2	10	0.6	2	10
S4	-0.02	0	19	-1.1**	-3	31	0.48	1	11
S5	0.98	3	6	-0.47***	-1	22	1.51	3	4
S6	1.13	3	5	0.77	2	8	0.05	0	16
S7	0.45	0	15	-0.15	0	18	-0.75	-2	26
S8	1.5	4	2	-0.94***	-3	30	1.27	3	6
S9	0.3	0	16	-0.77	-2	26	-0.1	0	19
S10	-1.47	-4	32	-2.18	-5	34	-1.43	-4	32
S11	1.3	3	4	0.63	2	9	0.62	2	9
S12	-1.16	-3	30	-0.63	-1	23	-0.46	-1	24
S13	-1.3	-3	31	-0.63	-1	24	0.73***	2	8
S14	0.74	1	12	0.47	1	12	-0.52*	-2	25
S15	1.66***	5	1	-1.25**	-4	32	-0.05**	0	18
S16	0.48**	1	14	1.55	3	4	1.31	3	5
S17	0.82	2	8	1.71*	4	2	0.9	2	7
S18	-0.07	0	20	-0.93	-2	28	-0.26	0	20
S19	0.89	2	7	0.16	0	15	1.84***	5	1
S20	0.75	1	11	0.77	2	7	1.74*	4	2
S21	-0.88	-2	25	1.56***	4	3	-0.8	-2	27

Table 4 List of Q-statements and factor Q-Sort values associated with the three perspectives

Statement Number	Factor 1			Factor 2			Factor 3		
	Z-score	Score	Rank	Z-score	Score	Rank	Z-score	Score	Rank
S22	0.1	0	18	-0.31	-1	21	0.14	0	15
S23	0.81	2	10	1.24	3	5	1.54	4	3
S24	1.37***	4	3	-0.15	0	19	0.29	1	12
S25	-0.87**	-1	24	0.32	1	14	0.02	0	17
S26	-1.14	-3	29	-0.31*	0	20	-1.14	-3	29
S27	-1.05	-2	27	-0.93	-3	29	-1.37	-3	31
S28	0.52***	1	13	-0.77	-2	27	-0.45	-1	23
S29	-0.77	-1	23	0.48***	1	11	-0.84	-2	28
S30	-0.27	-1	21	-0.01	0	17	0.27	1	13
S31	-0.92*	-2	26	2.03***	5	1	-1.53*	-4	33
S32	-0.69***	-1	22	1.09*	3	6	0.27*	1	14
S33	-1.54	-4	33	-1.55	-4	33	-1.2	-3	30
S34	-1.08**	-2	28	0	0	16	-0.37	-1	22

Note:1. The statements were written down in plain language to make the less-educated older adults to comprehend

2. Factor Q-sort values were identified by Q-sort factor analysis and indicated that the statements ranked from +5 (most agree) to -5 (most disagree)

3. Significance of Distinguishing Statements: $^{*}P<0.05$, $^{**}P<0.01$, $^{***}P < 0.001$
